# How should the law respond to emerging infectious diseases: China’s experience and considerations in containing COVID-19

**DOI:** 10.7189/jogh.14.03028

**Published:** 2024-06-14

**Authors:** Chenyu Yang, Xuan Li

**Affiliations:** Law School of Southeast University, Nanjing, China

Emerging infectious diseases significantly threaten global health in contemporary times [1]. The World Health Organization (WHO) publication Prioritizing Diseases for Research and Development in Emergency Contexts lists the most serious infectious diseases currently endangering humanity. Among them is Disease X, signifying the potential risk of a severe international epidemic caused by a pathogen currently unknown to cause human disease [2]. Since the public health emergency of international concern system was incorporated into the International Health Regulations in 2005, seven declarations of Public Health Emergency of International Concern have been declared as of 2022. Notably, three of these declarations resulted from emerging infectious diseases (although this count may be influenced by inconsistent criteria for defining emerging infectious diseases). These include the Influenza A (H1N1) pandemic, Ebola haemorrhagic fever, and coronavirus disease 2019 (COVID-19). The global impact of COVID-19, the emergence of pneumococcal pneumonia, and its aftermath have instilled fear, concern, and anxiety across the globe [3]. Above all, it underscored the urgent need to fortify the regulation and management of emerging infectious disease risks within the global public health governance framework.

In late December 2019, a previously unidentified coronavirus surfaced in Wuhan, China, triggering a devastating outbreak that rapidly spread to numerous Chinese cities and subsequently worldwide [4]. The initial outbreak of COVID-19 in China served as a litmus test for the efficacy of the nation’s infectious disease control system, developed in response to the 2003 Severe Acute Respiratory Syndrome (SARS) outbreak. China’s response to the challenges posed by COVID-19 presents a case study encompassing legal frameworks, administrative mechanisms, and governance. This narrative shares similarities with other nations while exhibiting distinctive characteristics unique to China. Subsequently, China plans to amend the Law of Prevention and Treatment of Infectious Diseases to rectify deficiencies observed in the practical application of COVID-19 prevention and control measures. This initiative aims to distil valuable experiences and institutionalise them into law, enhancing the nation’s preparedness to combat future outbreaks of emerging infectious diseases effectively. These legislative measures should serve as a blueprint for China’s future response to newly emerging infectious diseases, offering valuable insights and templates for the legal handling global public health emergencies.

## ADOPTING A PRESUMPTION OF GUILT APPROACH IN PREVENTING EMERGING INFECTIOUS DISEASES

China’s response to COVID-19, while faster than the reaction to the 2003 SARS outbreak, has faced criticism for its perceived delayed actions [5]. The first case in Wuhan surfaced as early as 8 December 2019, indicating human-to-human transmission by mid-December. Nonetheless, effective prevention measures commenced on 20 January 2020, when the State Council announced the enforcement of mandatory reporting for COVID-19 as a Class B disease, invoking strict measures akin to those applied for a Class A pathogen. Unfortunately, this coincided with the bustling Lunar New Year travel season, the great Chinese migration, leading to a nationwide COVID-19 outbreak. Political considerations, such as the confluence with Wuhan and Hubei provincial two sessions and pivotal annual political events in China, played a role in delaying or cancelling these events, facing substantial public and political pressure. Simultaneously, the absence of a distinct legal mechanism to tackle emerging infectious disease risks separate from established infectious diseases. There are various unavoidable obstacles in addressing newly emerging infectious diseases. It is crucial to recognise that the methods of legal regulation for such diseases are not exclusive to individual countries but rather require a global effort in development and resolution.

### The rights-based model of risk prevention and control

The management of communicable diseases varies globally, with each country designating a government agency responsible for curtailing their spread and incidence under the Communicable Disease Control and Prevention Act or equivalent special acts [6]. China’s infectious disease prevention and control approach operates under the notifiable diseases model, delineating clear legal frameworks governing the government’s response to infectious diseases (**Table 1**). While this model ensures a comprehensive system for collecting, analysing, identifying, and responding to known infectious diseases, emerging infectious diseases prompt only reporting responsibilities by the local government to the authoritative body (as the Law of Prevention and Treatment of Infectious Diseases, where the National Health Commission of the State Council oversees public health). Subsequently, the central authorities integrate the new infectious disease into the management of statutorily identified infectious diseases, allowing local authorities to implement effective prevention and control measures. This approach embodies the concept of prevention concerning emerging infectious diseases, wherein the potential harm requires substantial evidence, obligating the government to initiate preventive measures to contain the disease’s spread proportionately. While this structure prevents potential abuse of power by local governments, safeguarding citizens’ rights and freedoms, it risks missing the crucial golden period for epidemic prevention and control. Consequently, based on the experiences of the COVID-19 outbreak, amendments propose a suspicion-based model for risk prevention and control of emerging infectious diseases. This model dictates that upon identifying a new infectious disease with potential transmission risk, all suspected risks must be treated as definite dangers, ensuring prompt preparation of preventive and control measures to avoid missing the golden period.

**Table 1:** Amendments and clarifications related to the legal provisions on the prevention and control of infectious diseases in China

	Law of Prevention and Treatment of Infectious Diseases (2013)	First draft for soliciting comments (2020)	Second draft for soliciting comments (2023)	Explanations
Legal definition of emerging infectious diseases	No explicit provision exists, but ‘infectious diseases of unknown origin with sudden onset’ is adopted.	The usage includes ‘clustered diseases of unknown origin with characteristics of infectious disease outbreaks’ and ‘emerging infectious diseases.’	‘Outbreaks of infectious diseases of unknown origin’ and ‘emerging infectious diseases.’	Addition of regulatory details.
The subject of early warning	The health administrative department of the State Council and provincial-level people’s governments in China have the authority to issue early warnings.	County-level and higher-level people’s governments have the authority to issue early warnings.	County-level and higher-level people’s governments have the authority to issue early warnings and enjoy the right to provide interpretations.	Decentralisation of early warning authority.
Surveillance reports on emerging infectious diseases	No relevant provisions.	Web-based reporting within two hours.	Web-based reporting within two hours.	Addition of regulatory details.
Quarantine of emerging infectious diseases	Measures to establish it as a legally communicable disease.	If deemed necessary by the county-level and higher-level people’s governments after assessment, pre-emptive quarantine measures can be implemented.	Provisions have been added to allow higher-level authorities to revoke quarantine measures.	Provisions regarding quarantine measures for newly emerged infectious diseases have been added.

The concept of clustered diseases of unknown causes with epidemic characteristics has been introduced alongside emerging infectious diseases. Generally, infectious diseases classified as emerging share common traits – whether viral or bacterial – including unpredictability, high morbidity, the potential for rapid case escalation, and substantial societal impact. China’s methodology for identifying emerging infectious diseases accentuates this point [7]. Clustered diseases of unknown causes encompass illnesses exhibiting similar clinical manifestations, population clustering, epidemiological correlations, and severe health impacts without identifying novel pathogens. For instance, COVID-19 was initially regarded as unexplained pneumonia and fell under clustered diseases of unknown causes. The rationale behind introducing the concept of aggregated diseases of unknown origin is to avoid potential delays in epidemic prevention and control that may arise from solely categorising diseases as new infectious diseases. Incorporating both concepts aims to address challenges in promptly characterising new infectious diseases at the onset of their discovery.

**Figure:**
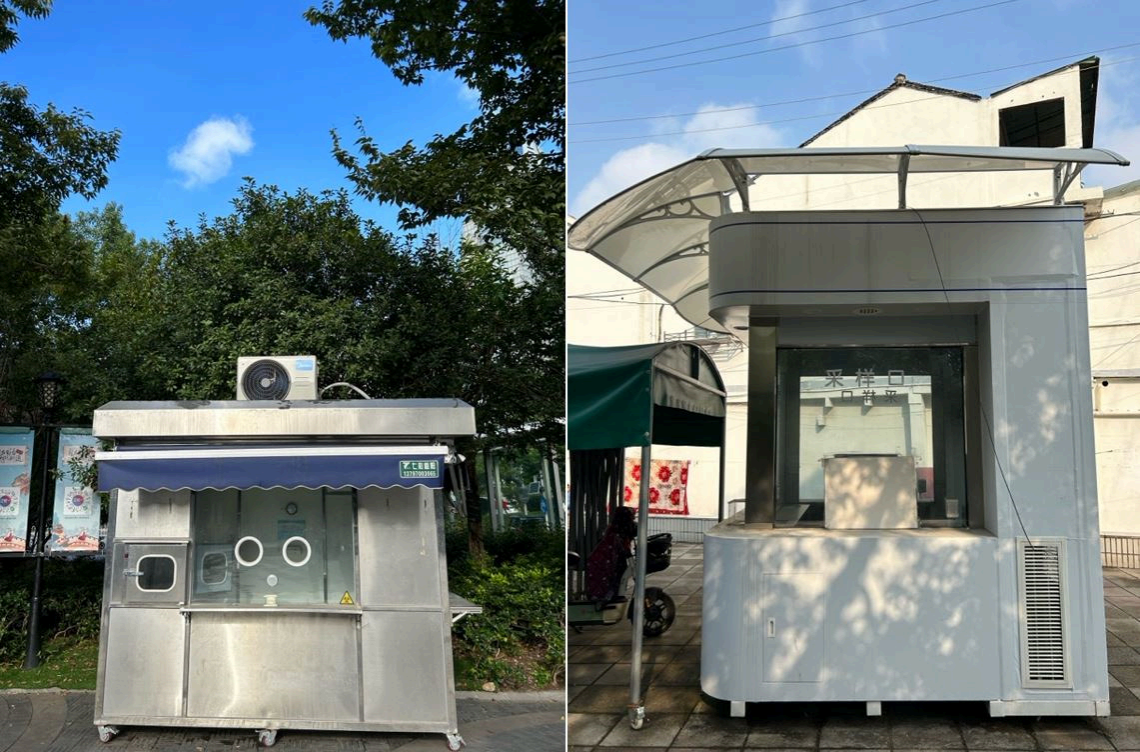
Photo: Abandoned testing booths post-COVID-19 outbreak. The left is taken in Wuxi, Jiangsu Province, China, and the right is taken in Wuhan, Hubei Province, China. Taken by the author Chenyu Yang.

Additionally, the authority to identify emerging infectious diseases and unexplained aggregated diseases has been delegated to the government in the affected area, diverging from the central government’s prior oversight. In the past, the power structure was hierarchical, with local governments and disease prevention and control institutions in epidemic areas receiving infectious disease alerts issued by the State Council health administrative departments or by provincial, autonomous region, and municipal governments. They were required to adhere to infectious disease prevention and control plans and implement corresponding preventive and control measures. This back-and-forth exchange of information through multiple departments and cumbersome procedures inevitably consumed a significant amount of time, which is crucial for the epidemic area. Local governments and disease prevention and control institutions in epidemic areas no longer need to await orders from higher-level departments. Instead, they possess greater autonomy and emergency response authority. Once an epidemic alert is issued, the local government and disease prevention and control institutions in the epidemic area immediately activate emergency plans and implement swift and effective preventive and control measures. They have direct monitoring and assessment capabilities for the epidemic and can adjust response strategies in real-time according to the situation.

This shift essentially relocates the primary responsibility for preventing and controlling emerging infectious diseases from the central government to the local government. Compared to the central government, local authorities possess inherent advantages in promptly acquiring information and responding rapidly to emergencies. They can swiftly mobilise local experts for early epidemic prevention and control. Acknowledging the varying capacities of local governments in identifying and assessing new infectious diseases – and the potential for misjudgement – the ‘draft for comments’ mandates immediate reporting by local governments to higher-level authorities for validation. This empowers higher decision-making bodies to verify the accuracy of these identifications. In assessing information communication, an information-sharing mechanism is used between government departments at all levels to ensure that information on epidemics is transmitted and shared on time.

### Principle of *prima facie* evidence

Determining an emerging infectious disease necessitates adherence to a *prima facie* evidence rule [8]. The principle of prima facie evidence is generally used to describe how a situation appears on initial observation. In the legal system, *prima facie* is commonly used to refer to either a piece of evidence that is presumed to be true when first viewed or a legal claim in which enough evidence is presented to support the claim’s validity. In public health, *prima facie* evidence can be applied to formulate initial hypotheses and assessments regarding emerging infectious diseases. During the early phases of an outbreak, when the pathogen and its characteristics are not yet comprehensively understood, preliminary speculations and judgments can be made by relying on existing visible indicators and prior experience. In the initial stages of an emerging outbreak, knowledge about the disease remains largely uncertain, including the pathogen source, transmission mode, infectiousness, and clinical features. Early indications suggest that the overall morbidity and mortality rates may not significantly differ from those of seasonal influenza. Identifying an emerging infectious disease often requires laboratory testing for novel pathogens and epidemiological investigations to ascertain infectivity. However, this process is time-consuming and not conducive to early outbreak prevention and control. Given the high probability of association between new clinical characteristics and new pathogens, presumptions based on facts can expedite identification. For instance, although pneumococcal pneumonia imaging aligns with typical viral pneumonia features, the standard clinical treatment for viral pneumonia has proven ineffective, suggesting a distinct specificity independent of other viral pneumonia and indicating a potential novel infectious disease [9].

## TIMELY WHISTLEBLOWING FOR EARLY WARNING OF EMERGING INFECTIOUS DISEASES

After the 2003 SARS epidemic, China allocated CNY 700 million to establish a nationwide direct network reporting system for infectious diseases. This network covered all Centres for Disease Control and Prevention (CDC) and 98% of medical agencies above the county level, allowing these agencies to report directly to China CDC in real-time within hours. Despite being hailed as one of the world’s premier disease control programs, the system failed to promptly detect and issue an alert for the COVID-19 outbreak [10]. We must acknowledge that the demand for an immediate response to sudden and newly emerging infectious diseases is a challenging requirement. However, this also underscores an important fact. The most advanced disease control and early warning systems worldwide can experience delays. This underscores the significant challenge that the sudden and urgent nature of emerging infectious diseases presents to the existing alert mechanisms and systems established by human society. We should focus on analysing and reflecting upon these challenges to enhance our preparedness and response capabilities for future crises. The issue of early warning encompasses multiple facets.

### The dilemma of inflated focus on infectious disease alerts: Novel outbreaks or statutory designation?

The early-warning mechanism focuses on statutory infectious diseases, overlooking emerging ones (**Table 1**). Most infectious disease prevention and control legislation neglects to define specific roles for each level of government and lacks delineation on how these government bodies will coordinate vertically during emergencies [11]. For statutory infectious diseases, officially determined diagnostic criteria mandate reporting and intervention measures upon meeting these criteria. However, there is a gap in institutional design for emerging infectious diseases, requiring accurate aetiological assessment before reporting. The absence of standardised surveillance protocols for emerging infectious diseases hampers the direct reporting system’s capacity within the infectious disease network. Consequently, during early surveillance, COVID-19 was categorised as typical viral pneumonia, commonly occurring annually, leading to its omission in the direct reporting system. Hence, local governments are effectively granted greater discretion in reporting emerging infectious diseases without immediate obligation to report outbreaks. Wuhan mayor invoked Article 3 of the Law of Prevention and Treatment of Infectious Diseases to attribute the lack of public disclosure of emerging infectious diseases to higher-level approval. As leaders of the local government, the actions of the mayor of Wuhan were reasonable and deserving of recognition before the implementation of new regulations. However, this also underscores the significance of the legal impact on the decision-making process of government leaders at the regional level in epidemic-affected areas.

### Assignment of authority for early warning decisions

Under the Law of Prevention and Treatment of Infectious Diseases, early warning decisions lie with the National Health Commission or higher-level provincial governments, not the government where the outbreak occurs. According to the Infectious Disease Prevention and Control Law, only the health administrative departments of the State Council and the provincial-level people’s governments have the authority to issue alerts for infectious diseases. On the other hand, the Emergency Response Law stipulates that the people’s governments at or above the county level can issue alerts corresponding to their respective levels. The National Public Health Emergency Response Plan specifies that health administrative departments at all levels of government should issue timely warnings based on monitoring information. These laws, enacted by the Standing Committee of the National People’s Congress, exhibit inconsistent provisions. Moreover, the lower-level emergency response plans directly authorise the health administrative departments. This issue cannot be resolved simply by applying the principles of special laws overriding general laws or new laws superseding old laws. This raises questions about a rational distribution of early warning authority within a hierarchical bureaucratic structure [12]. Given the extensive emergency measures and economic costs following early warnings, the law characterises early warning as a political judgment rather than a medical professional judgment. It mandates higher-level government entities to make well-considered decisions by integrating political, economic, and scientific factors to prevent public panic and unrest due to potentially erroneous warning information.

### Difficulties in the dissemination of early warning information

Early warning hinges on access to information to comprehend the situation’s development. Information on infectious disease outbreaks follows a funnel structure, ascending through layers until reaching the authority responsible for issuing warnings. Local governments, at the frontline of new infectious disease prevention and control, possess firsthand information about epidemics. However, faced with uncertain issues (a pandemic or common viral pneumonia), they report positive news and withhold negative information, fearing repercussions for lower-level government failures. Reporting to higher levels might be perceived politically as transferring pressure upward to evade responsibility. Consequently, higher-level authorities vested with early warning authority might not receive timely information for appropriate early warning decisions.

### China’s legal response to timely warning: Addressing the need for prompt alerts

China took immediate action by convening experts from various fields, such as medicine, law, administrative management, and public health. Under the coordinated direction of the government, discussions were held to solicit feedback on the draft of the new law regarding newly emerging infectious diseases, focusing on addressing and improving these issues.

First, it establishes a unified information monitoring and sharing mechanism, promoting infectious disease surveillance information sharing across departments and regions. It standardises information-sharing processes and enables interoperable clinical medical and disease control information sharing. This involves the establishment of sentinel surveillance of emerging infectious diseases, expanded monitoring of infectious disease symptoms, collection of infectious disease syndromes, and an aetiology surveillance network.

Second, the draft introduces a whistleblower system to compensate for administrative surveillance and early warning deficiencies and harness public involvement in emerging infectious disease surveillance. This system incentivises and exempts individuals reporting infectious disease outbreaks from liability to protect whistleblowers. Lastly, it amalgamates immediate prevention and control measures with the early warning mechanism by devolving early warning power to county-level and above people’s governments. When the government monitors an emerging infectious disease outbreak risk in the outbreak location, it can issue direct warning information to the public. Using non-coercive means like guidance and advice enhances public awareness and takes moderate preventive measures such as risk warnings, advising mask-wearing and social distancing. Simultaneously, it implements targeted measures based on the risk level of the emerging infectious disease, including patient isolation, contact tracing, environmental sanitation, and medical observation.

## IMPLEMENTING REASONABLE QUARANTINE MEASURES IN CONTROLLING THE SPREAD OF EMERGING INFECTIOUS DISEASES

### Representation of the rationality crisis in quarantine measures

Throughout human history, the defeat of most plagues did not rely on drugs or vaccines but rather on the simple practice of severing transmission routes. In the context of the recent pneumonia epidemic, the most effective approach to prevent and control new infectious diseases has been to halt the transmission route. Restricting the personal freedom of patients, suspected cases, and close contacts has emerged as the most direct and efficient strategy to sever transmission routes. On 23 January 2020, authorities in Wuhan implemented a decision to enforce a city-wide lockdown, suspending all incoming and outgoing traffic, imposing social distancing measures on over 10 million people, confining residents to their homes, and providing essential supplies through government channels [13]. The allocation of resources and daily necessities for individuals undergoing quarantine measures poses a major challenge and a difficult decision for China. It requires careful consideration not only of the practical needs of the quarantined population but also the emotional impact of isolation on individuals, as well as the international scrutiny and public discourse surrounding the potential limitations on personal freedoms associated with quarantine measures. The decisive decisions taken by the Chinese government regarding quarantine measures, along with the robust protection of human rights for individuals in isolation, have resulted in notable accomplishments. China successfully curbed the transmission route by implementing robust mass quarantine and isolation measures. This resulted in zero reported indigenous cases nationwide in less than two months (by 18 March 2020), ultimately allowing Wuhan to lift its closure. China’s effective epidemic prevention and control strategies emerged as a valuable model for other nations. However, what sets this situation apart is that, based on the assessment of the severity of COVID-19, China has implemented quarantine measures of an unprecedented scale and severity. This has triggered a critical examination of the rationality behind the stringent quarantine measures.

Indeed, many countries have implemented similar quarantine measures in response to the COVID-19 pandemic. As the virus has spread, numerous countries have implemented measures such as travel restrictions, lockdowns, home isolation, and centralised quarantine to contain the spread of the virus and protect public health. However, criticisms of these quarantine measures mainly focus on the following aspects: restrictions on individual freedoms, the potential for increased infection rates among uninfected individuals due to strict regional isolation measures, significant infringement on the rights of vulnerable groups during isolation, adverse effects on individuals’ mental health due to prolonged periods of isolation, leading to severe psychological stress responses, and the possibility that enforced isolation and travel restrictions may perpetuate stereotypes and biases against certain individuals based on their appearance, race, or ethnic background, rather than relying on scientific facts [14].

### A new perspective on the rationality crisis in quarantine measures: Bringing the legality of isolation back to its rational essence

Quarantine originally referred to the selective self-isolation or isolation of individuals suspected of being carriers of infection. However, a new term – mass quarantine – is now emerging, which primarily denotes the government’s compulsory isolation of populations to prevent the spread of disease outbreaks [15]. Quarantine encompasses two interpretations – isolating diagnosed/suspected patients to prevent transmission and provide medical care and isolating close contacts or individuals in affected areas to assess potential infection and restrict movement. Quarantine, in a broader sense, encompasses patient isolation, isolation of close contacts, lockdowns in affected areas, work suspensions, and traffic controls, constituting the most restrictive preventive measures on civil rights within the infectious disease control system. The law of China outlines stringent application conditions, limiting quarantine measures strictly to Category A infectious diseases and specifically listed diseases in Category B. These include five highly contagious diseases – plague, cholera, SARS, pulmonary anthrax, and highly pathogenic avian influenza. For emerging infectious diseases necessitating isolation and quarantine, approval from the State Council via the Ministry of Health is mandatory before declaration and implementation. This conversion process is required to classify newly discovered infectious diseases as legally defined ones before instituting isolation and quarantine measures. This approach centralises the decision-making on quarantine measures for patients with emerging infectious diseases to the State Council, reflecting the rigour of restricting personal freedoms. Consequently, the lack of a clear legal basis for quarantine measures in the early stages of newly emerging infectious diseases, particularly for patients and close contacts, hinders the timely epidemic control due to these constraints.

The complex process involved in quarantine decisions and the limitations imposed on decision-makers is based on careful considerations of the legality of quarantine measures. However, one of the underlying causes of the rationality crisis lies in the overly burdensome legal procedures and the absence of specific guidance in legal frameworks. This represents a new interpretation of the rationality crisis, highlighting that a measure’s legality does not necessarily guarantee its rationality. The assessment of a measure’s legality and rationality are based on different criteria. While the legality review of most measures is guided by legal principles and values, including considerations of rationality, it is often heavily influenced by factors such as politics, ideology, past legal frameworks, and current national policy directions, which may not have a strong alignment with rationality.

China’s resolution of the rationality crisis regarding quarantine measures is primarily demonstrated through its handling of specific issues related to the COVID-19 pandemic. To address the lack of initiative by local governments in the outbreak area before newly emerging infectious diseases are officially managed as statutory infectious diseases, the draft proposal empowers local governments extensively to prevent and control new infectious diseases. This includes implementing isolation measures for patients with new infectious diseases and their close associates. Article 50 of the draft empowers people’s governments at or above the county level to directly implement quarantine measures upon detecting an emerging infectious disease (**Table 1**). This provision aligns with the International Health Regulations requirements. Annex 1 of the International Health Regulations necessitates local community-level or primary public health response-level abilities to implement preliminary control measures immediately. The draft interprets preliminary control measures to encompass the most restrictive freedom rights measures, such as isolation and quarantine. From the perspective of effectively preventing emerging infectious diseases, empowering local governments to isolate or quarantine patients and potentially infected individuals upon detecting an emerging infectious disease becomes crucial. Admittedly, this approach does not eliminate the possibility of inappropriate restrictions on false positives or later evaluations of an overreaction to an emerging infectious disease outbreak. Yet, this is inherently an iterative process and concerns excessive intrusion into personal liberty that can be mitigated through ongoing collection and analysis of infectious disease information. Continuous assessment of infectiousness and pathogenicity allows timely adjustments to the scope and duration of isolation and quarantine measures. To this end, the National Health Commission has issued ten editions of the Prevention and Control Plan for COVID-19 and the Treatment Plan for COVID-19 as normative guidelines for epidemic prevention and control across regions. These documents aim to update pathogenic characteristics, clinical symptoms, treatment plans, criteria for quarantine measures, contact isolation timelines, and criteria for new infectious diseases, reflecting a dynamic and responsive approach.

It is important to provide an explanation and differentiate between three concepts – legality, rationality, and legal normativity (narrow sense legality). Pure legality represents the ideal state where rationality and legal normativity are harmonised, reflecting the optimal expression of a measure. Rationality involves evaluating a measure based on its value or utility. Legal normativity pertains to whether a measure conforms to the provisions of existing legal norms. Consequently, previous discussions on the rationality of quarantine measures have often been conflated with discussions on legality [16,17]. By examining the actual circumstances of China’s quarantine measures, a new perspective emerges on the rationality crisis, where the legal normativity of these measures can pose a barrier to achieving rationality in certain situations. Therefore, overcoming the rationality crisis of quarantine measures necessitates the establishment of robust legal frameworks for these measures and striking a balance between legality and rationality using the principle of proportionality.

## CONCLUSIONS

Taking the COVID-19 pandemic as the research background, we conducted an experiential analysis of China’s risk prevention measures and management strategies in the face of emerging infectious diseases. We aimed to construct a comprehensive and universal legal framework for risk prevention and control of newly emerging infectious diseases from a legal perspective. The aim of this framework is to provide practical legal guidance for future global public health crises triggered by newly emerging infectious diseases, highlighting the crucial role of law in the global health era. Due to the sudden and unpredictable nature of such events, it is impossible to predict the timing of the next global pandemic caused by a new infectious disease, let alone eliminate the occurrence of such events from the source. However, efforts will certainly be made to prevent similar adverse consequences from happening again. The key lies in adopting a risk prevention rather than a danger prevention approach in dealing with future outbreaks of new infectious diseases. This involves using legally binding and effective regulatory texts to address the harms caused by global public health emergencies in a more targeted and efficient manner. Instead of wasting valuable time waiting for pathogen research results or developing medicines and vaccines, the focus should be on early warning and post-outbreak risk control.
